# Living-donor kidney transplantation: comparison of robotic-assisted versus conventional open technique in obese recipients

**DOI:** 10.1007/s00345-026-06241-3

**Published:** 2026-02-18

**Authors:** Alice Rondot, Stephan Levy, Jérémy Mercier, Anne Sophie Bajeot, Arnaud Del Bello, Nassim Kamar, Xavier Gamé, Nicolas Doumerc, Federico Sallusto, Thomas Prudhomme

**Affiliations:** 1 Department of Urology and Kidney Transplantation, Toulouse University Hospital, TSA 50032 Rangueil Hospital, 31059 Toulouse Cedex 9, France; 2 Department of Urology and Transplantation, Edouard-Herriot University Hospital, Hospice Civils de Lyon, Lyon, France; 3 Department of Nephrology and Organ Transplantation, Toulouse University Hospital, Toulouse, France

**Keywords:** Living donor kidney transplantation, Robotic-assisted kidney transplantation, Open kidney transplantation, Delayed graft function, Obese recipients

## Abstract

**Purpose:**

The objective was to compare the intraoperative, postoperative and functional outcomes of obese recipients undergoing robot-assisted living donor kidney transplantation (RAKT) compared to conventional open kidney transplantation (OKT).

**Methods:**

A retrospective analysis of living donor’s kidney transplantation performed in a tertiary French academic center between January 2012 and January 2025 was performed. Only recipients who were obese (BMI ≥ 30 g/m^2^) at the time of transplantation were included.

**Results:**

A total of 86 patients were included in our study, including 46 patients in the RAKT group and 40 patients in the OKT group. The two groups were comparable except a higher rate of previous abdominal surgery in the OKT group (65% versus 28%; *p* = 0.001). Early postoperative complications and delayed graft function were similar between the 2 groups. One death occurred on postoperative day 15 in the OKT group due to cardiac arrest. Two open conversions occurred in the RAKT group: one due to active bleeding and one due to intraoperative venous thrombosis. Median length of hospitalization was significantly longer in the OKT group (12 versus 9 days, *p* = 0.001). The rate of surgical reintervention after POD 90 was significantly higher in the OKT group (37.5% versus 6.5%, *p* = 0.003). 1- and 3-patient and graft survival were comparable between the RAKT and OKT cohorts.

**Conclusions:**

Our outcomes confirms the safety and efficacy of robotic approach for living donor kidney transplantation in obese recipients.

## Introduction

Kidney transplantation is the best therapeutic option to offer to patients with end-stage renal failure, owing to greater survival rate and better quality of life in comparison with dialysis [1–4]. The conventional open kidney transplantation (OKT) technique was described by Kuss et al. [5] in the early 1950s. Currently, this surgical procedure is well codified and follows consistent principles, even though each team has its own technical characteristics [6, 7].

Over the last 30 years, the minimally invasive surgery has revolutionized surgical practice resulting in a rapid dissemination of the laparoscopic surgery. However, in the field of kidney transplantation, the technical difficulties in performing vascular anastomosis in the pelvis using laparoscopic instruments and two-dimensional vision has limited its expansion. The introduction of the da Vinci ^®^ robotic surgical system (Intuitive Surgical Inc, Sunnyvale, CA, USA) has filled the gap, enabling the precise intracorporeal vascular anastomosis required for kidney transplantation [8]. Several teams have described the standardized technique of robot-assisted kidney transplantation and confirmed that surgical procedure is safe [9, 10]. 

The World Health Organization defines obesity as having a body mass index (BMI in kg/m^2^) of ≥ 30 kg/m^2^. In 2016, more than 1.9 billion adults were overweight and over 650 million were obese [11]. In 2017, in France, 23% of dialysis patients were obese [12]. Obesity is a risk factor of chronic kidney disease [13] and the prevalence in the dialysis patient population is constantly increasing [14].

Although the benefits of kidney transplantation in obese patients are well established [15], the postoperative complications are more frequent in the obese population, such as parietal complications, eventration, infection, hematoma, deep vein thrombosis or pulmonary embolism [16].

Despite the RAKT technique has been standardized and its feasibility demonstrated in several clinical scenarios, there is still a lack of data directly comparing perioperative and postoperative outcomes between RAKT and OKT in the specific obese population. To fill this gap, the aim of this study was to compare intraoperative, postoperative and functional outcomes of obese recipients undergoing living donor RAKT versus open kidney transplantation (OKT), in a tertiary French academic center.

## Methods

### Study design and patient’s selection

A retrospective analysis of all living donor kidney transplantation performed at the University Hospital of Toulouse, France, between January 2012 and January 2025 was conducted. We collected all consecutive cases performed in obese recipients (BMI ≥ 30 kg/m^2^) during this period. The exclusion criterion for a RAKT were the following: (a) medical history of complex abdominal surgeries, (b) severe atherosclerotic plaques at the level of external iliac vessels at the preoperative computed tomography angiogram, (c) prior bilateral kidney transplantation. For this study, in order to mimic the RAKT conditions, the exclusion criterion in the OKT cohort including (a) orthotopic KT, (b) KT on vascular prosthesis, (c) severe atherosclerotic plaques at the level of external iliac vessels at the preoperative computed tomography angiogram, (d) prior bilateral kidney transplantation. Thus, all recipients in the OKT group were eligible for a robotic-assisted kidney transplantation.

### Surgical procedure

The surgical procedure were performed by three different surgeons.

#### Robotic-assisted kidney transplantation

RAKT were performed using the da Vinci Xi Surgical System (Intuitive Surgical Inc., Sunnyvale, CA, USA) in a four-arm configuration, with a 30° lens and a 25° Trendelenburg tilt. The cases were performed by two surgeons. In our institution, the RAKT technique followed the principles of the Vattikuti-Medanta technique, using a transperitoneal approach [8–10, 17–20].

#### Open kidney transplantation

OKTs were performed following conventional retroperitoneal technique via Gibson incision [6]. The cases were performed by three different senior surgeons.

### Immunosuppression

All patients received triple immunosuppression therapy, including calcineurin inhibitor, steroids and either mycophenolic acid or an mTOR inhibitor. Induction was either basiliximab or antithymocyte globulin, accord to immunological risk.

### Study variables

Donor-, graft- and recipient-related data’s, intraoperative outcomes, early post-operative (≤ day 90) complications and functional outcomes as well as follow-up outcomes were retrospectively collected.

Total operative time was calculated from case start (incision time) until case end (closure). Delayed graft function (DGF) was defined as the need of dialysis in the first week following KT [21]. The estimated glomerular filtration rate (eGFR) calculation was performed using the Chronic Kidney Disease Epidemiology Collaboration formula [22]. Postoperative surgical complications were reported according to modified Clavien-Dindo system [23] and high-grade postoperative complications were defined as Clavien-Dindo grade ≥ 3.

### Statistical analysis

Quantitative data were expressed as medians with interquartile range (IQR) as well as range and were compared using the Mann–Whitney *U* test for nonnormally distributed variables. Qualitative data were expressed as numbers and percentage and were compared using chi-square and Fisher exact tests. Overall survival was estimated using the Kaplan–Meier method, and RAKT and OKT cohorts were compared via log-rank tests.

A *P* value of < 0.05 was considered statistically significant. Statistical analyses were performed using S PRISM v.10.1.1 (GraphPad Software Inc., La Jolla, CA, USA) and IBM SPSS v29 (IBM Corporation, NY, USA).^1^

## Results

### Baseline recipients- and graft-related characteristics

A total of 46 living donor RAKT were compared to 40 OKT. All recipients were obese (BMI ≥ 30 kg/m^2^). The baseline recipients and graft-related characteristics in the RAKT and OKT cohorts were reported in Table 1.

Both study groups were comparable regarding recipients’ median age, gender, median BMI, median Charlson score, previous kidney transplantation, pre-emptive status, median dialysis duration and ABO incompatible transplant proportion. A significantly higher proportion of recipients receiving OKT had undergone previous major abdominal surgery (65% versus 28.3% *p* = 0.001). The grafts characteristics’ including laterality, number of arteries and number of veins were comparable between the RAKT and OKT cohorts.

**Table 1:** Recipient and kidney graft characteristics

	Overall population *n* = 86	Robot assisted transplantation *n* = 46	Open transplantation *n* = 40	*p*
*Récipient characteristics*				
Recipient age (years) (median, IQR)	56.4 (45.1–61.4)	56.7 (44.2–61.6)	54.9 (45.5–61.3)	0.6
*Recipient sexe (n, %)*				
Male	62 (72.1%)	35 (76.1%)	27 (67.5%)	0.5
Recipient BMI (kg/m^2^) (Median, IQR)	32.0 (31.0–34.0)	33.0 (30.9–35.1)	32.0 (31.0–33.0)	0.1
Recipient Charlson comorbidity index (Median, IQR)	2.0 (2.0–3.0)	2.0 (2.0–4.0)	2.0 (2.0–3.0)	0.6
History of abdominal surgery (n, %)	39 (45.3%)	13 (28.3%)	26 (65.0%)	**0.001**
History of kidney transplantation (n, %)	11 (12.8%)	4 (8.7%)	7 (17.5%)	0.3
Preemptive kidney transplantation (n, %)	44 (51.2%)	20 (43.5%)	24 (60.0%)	0.1
Duration of dialysis for non preemptive transplantation (months) (Median, IQR)	12.0 (9.0–36.0)	12.0 (5.0–50.0)	14.0 (12.0–36.0)	0.3
ABO incompatible kidney transplantation (n, %)	30 (34.9%)	12 (26.1%)	18 (45.0%)	0.1
*Graft characteristics*				
Graft laterality (n, %)				
Left kidney	84 (97.7%)	44 (95.7%)	40 (100%)	0.5
Right kidney	2 (2.3%)	2 (4.3%)	0 (0%)	
Number of graft artery (n, %)				
1	75 (87.2%)	42 (91.3%)	33 (82.5%)	0.3
2	11 (12.8%)	4 (8.7%)	7 (17.5%)	
Number of graft vein (n, %)				
1	83 (96.5%)	46 (100%)	37 (92.5%)	0.1
2	3 (3.5%)	0 (0%)	3 (7.5%)	

### Intraoperative outcomes

The intraoperative outcomes of the RAKT and OKT cohorts are reported in Table 2.

The majority of KT were performed in the left iliac fossa. The median overall operative time was significantly longer in the OKT group (215.5 versus 140.0 min, *p* < 0.0001). Intraoperative major post-operative complications rate was similar in both study group (2.5% versus 2.2%, *p* = 0.9). Two (2.2%) open conversion occurred during RAKT, due to active bleeding and venous thrombosis.

**Table 2:** Intraoperative and early postoperative data, and early functional outcomes of kidney transplantations

	Overall population *n* = 86	Robot assisted transplantation *n* = 46	Open transplantation *n* = 40	*p*
Intraoperative data				
*Transplantation site (n, %)*				
Left iliac fossa	80 (93.0%)	44 (95.7%)	36 (90.0%)	0.9
Right iliac fossa	6 (7.0%)	2 (4.3%)	4 (10.0)	
Operative time (minutes) (Median, IQR)	170.0 (134.5-215.5)	140.0 (120.0-160.0)	215.5 (195.3–264.0)	**< 0.0001**
*Intraoperative complications (n, %)*				
Active bleeding	1 (1.2%)	1 (2.2%)	0 (0%)	0.9
Venous thrombosis	2 (2.3%)	1 (2.2%)	1 (2.5%)	
Surgical conversion and causes (n, %)	–	2: Intraoperative active bleeding and venous thrombosis	–	–
*Early Postoperative Data (Day 90)*				
Lenght of hospital stay (days) (Median, IQR)	10.0 (8.0–14.0)	9.0 (7.0–13.0)	12.0 (9.0-15.8)	**0.001**
Early postoperative complications (Clavien-Dindo classification) (n, %)				
*Grade 2*				0.9
Bleeding requiring transfusion	2 (2.3%)	1 (2.2%)	1 (2.5%)	
Wound infection	1 (1.2%)	1 (2.2%)	0 (0%)	
Urinary tract infection	6 (7.0%)	2 (4.3%)	4 (10.0%)	
*Grade 3a*				
Nephrostomy placement for urinary fistula	1 (1.2%)	1 (2.2%)	0 (0%)	
Radiologic drainage of lymphocele	1 (1.2%)	0 (0%)	1 (2.5%)	
*Grade 3b*				
Graft nephrectomy for : a) Venous thrombosis	3 (3.5%)	2 (4.3%)	1 (2.5%)	
Reoperation by laparotomy for:				
a) Renal vein plication	2 (2.3%)	1 (2.2%)	1 (2.5%)	
b) Evisceration	5 (5.8%)	2 (4.3%)	3 (7.5%)	
c) Subcapsular hematoma	1 (1.2%)	1 (2.2%)	0 (0%)	
Angioplasty with stenting of the external iliac artery for dissection	1 (1.2%)	0 (0%)	1 (2.5%)	
*Grade 4*				
Respiratory failure	1 (1.2%)	1 (2.2%)	0 (0%)	
Peritonitis due to graft abscess with colonic fistula	1 (1.2%)	1 (2.2%)	0 (0%)	
Grade 5	1 (1.2%)	0 (0%)	1 (2.5%) : Cardiac arrest on day 15	
Early major postoperative complications (Clavien-Dindo grade ≥ 3) (n, %)	17 (19.8%)	9 (19.6%)	8 (20.0%)	0.9
Early functional outcomes				
Delayed graft function (n, %)	14 (16.3%)	6 (13.0%)	8 (20.0%)	0.4
Postoperative serum creatinine (mg/dl) (Median, IQR)				
J7	1.8 (1.4–3.5)	1.6 (1.4–2.9)	2.3 (1.5–4.5)	0.1
J30	1.5 (1.3–1.9)	1.5 (1.3-2.0)	1.6 (1.3–1.9)	0.7
Postoperative estimated creatinine clearance (CKD-EPI 2009, ml/min/1.73m^2^) (Median, IQR)				
J7	35.5 (16.0-52.3)	42.5 (21.8–56.5)	24.0 (10.0-42.8)	**0.01**
J30	49.0 (37.0–57.0)	49.5 (37.0-63.8)	48.0 (36.0–54.0)	0.8
*Postoperative recipient hemoglobin (g/dl) (median, IQR)*				
J7	9.8 (9.0–10.6)	10.0 (9.1–11.2)	9.6 (8.8–10.4)	0.2

### Postoperative and early functional outcomes

An overview of the early postoperative outcomes after RAKT versus OKT is provided in Table 2. The median length of hospitalization (LOH) was significantly longer in the OKT group (12 versus 9 days, *p* = 0.001). Post-operative surgical complications and major post-operative surgical complications rates were similar between the RAKT and OKT cohorts (major post-operative surgical complications: 19.6% versus 20.0%; *p* = 0.9). One death occurred on postoperative day 15 in the OKT group, due to due to cardiac arrest.

There were no significant differences between RAKT and OKT regarding delayed graft function rate as well as in the serum creatinine and hemoglobin trajectories after transplantation. The median eGFR at POD 7 was significantly higher in the RAKT group (42.5 versus 24.0 ml/min/1.73m^2^; *p* = 0.01).

### Follow-up outcomes

Follow-up outcomes after RAKT versus OKT are shown in Table 3.

**Table 3:** Follow up data after robot assisted versus open kidney transplantation

	Overall population *n* = 86	Robot assisted transplantation *n* = 46	Open transplantation *n* = 40	*p*
*Follow up*				
Follow up duration (months) (Median, IQR)	47.5 (12.3–84.0)	24.4 (9.0-60.5)	89.0 (27.3-136.1)	**< 0.0001**
Surgical reinterventions related to kidney transplantation after day 90 (n, %)				
Eventration	7	2	5	**0.003**
Transplantectomy	8	1	7	
Marsupialization	1	0	1	
Serum creatinine at last follow up (mg/dl) (Median, IQR)	140.0 (110.0-195.0)	140.0 (110.0-180.0)	140.0 (105.0-200.0)	0.9
Graft survival (n, %)				
1 year	95.1%	97.7%	92.4%	0.8
3 years	86.5%	85.1%	86.5%	
Patient survival (n, %)				
1 year	96.3%	100.0%	92.4%	0.7
3 years	94.6%	95.8%	92.4%	

The median follow-up was significantly longer in the OKT group (24.4 versus 89.0 months, *p* < 0.0001). The rate of surgical reintervention after POD 90 was significantly higher in the OKT group (37.5% versus 6.5%, *p* = 0.003).

**Fig. 1:**
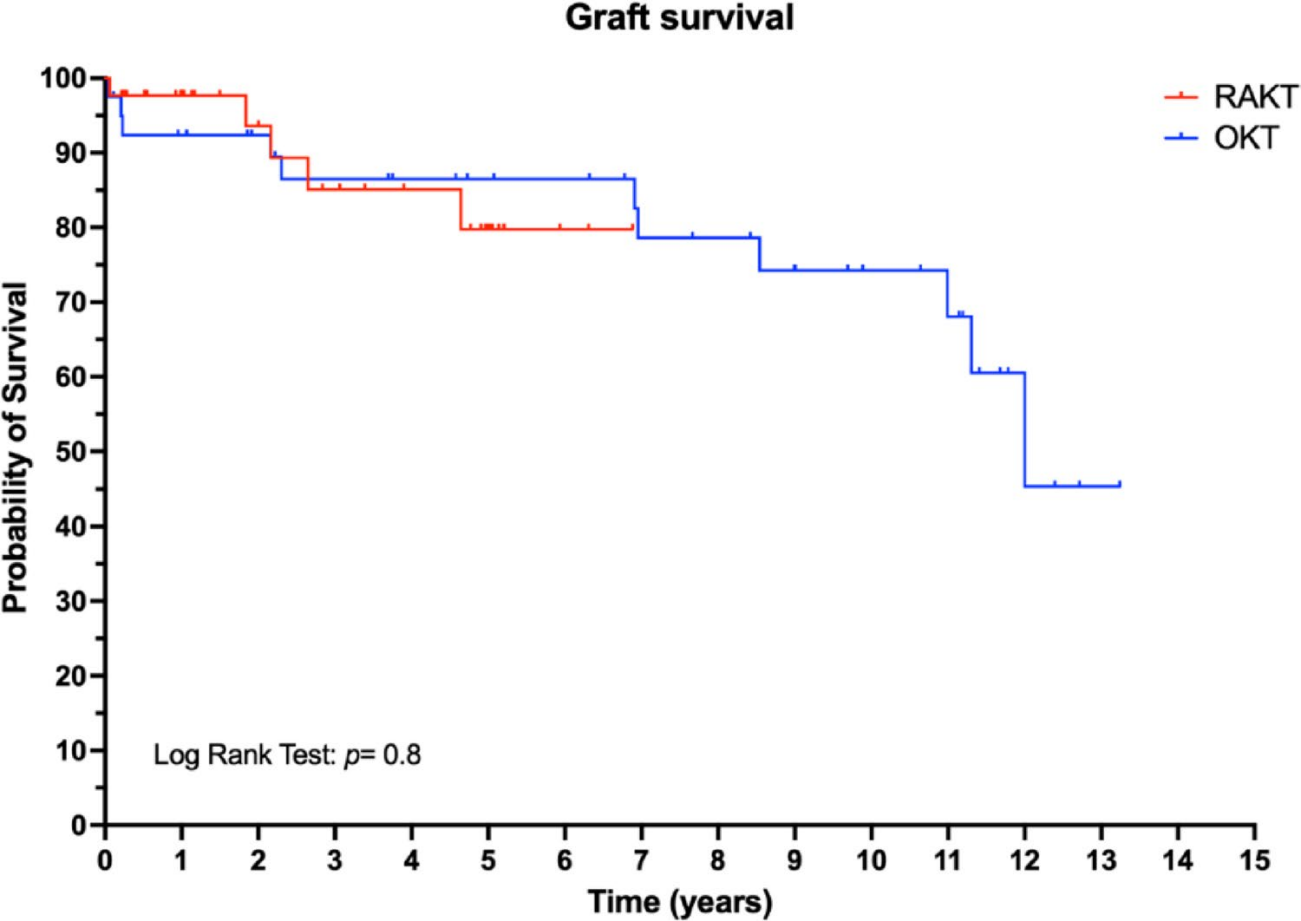
Graft survival

**Fig. 2:**
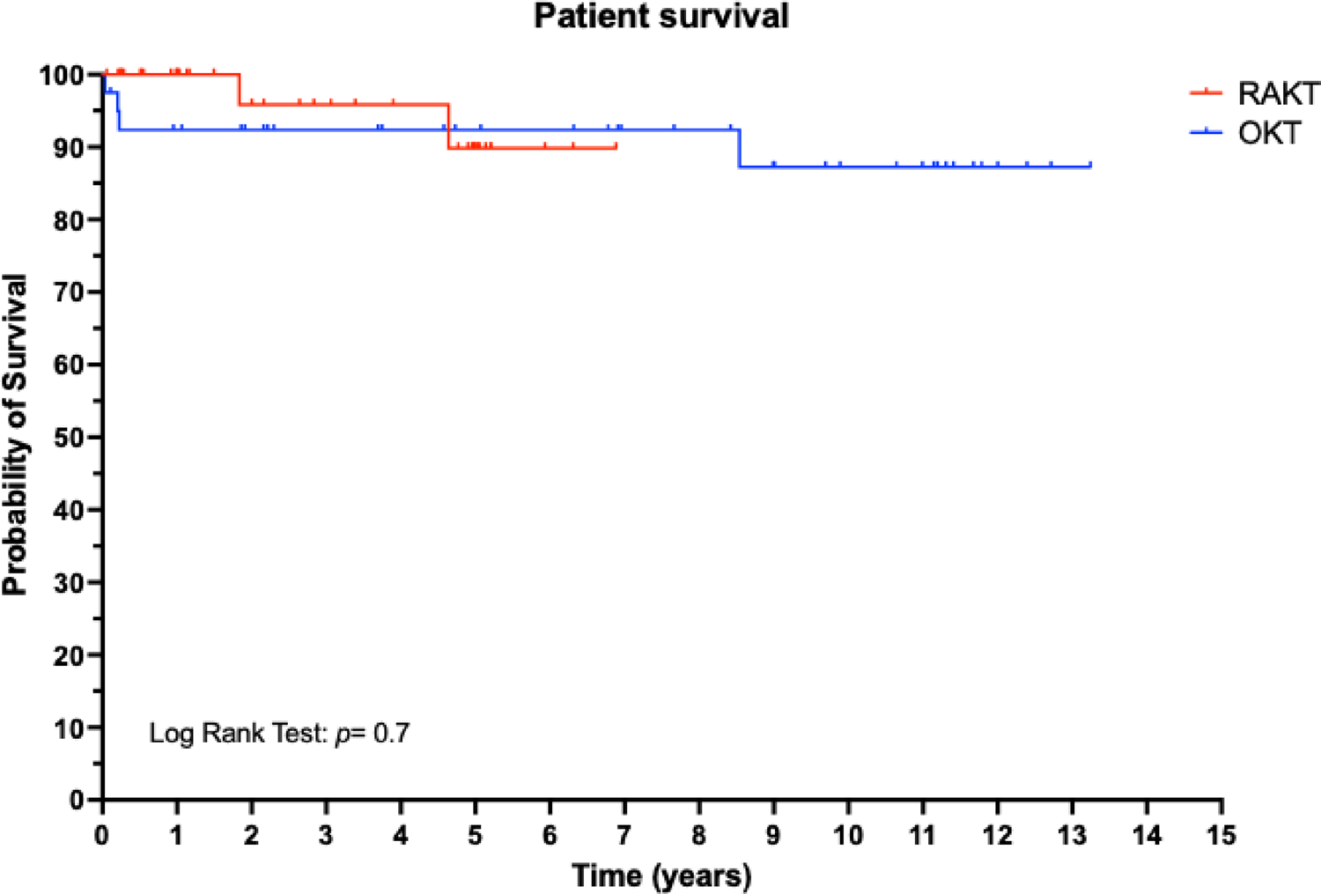
Patient survival

At last follow-up, the median serum creatinine was comparable in RAKT and OKT group. One and three-years patient and graft survival were comparable between the RAKT and OKT cohorts, Figs. 1 and 2.

## Discussion

During past 3 decades, minimally invasive surgery has increasingly permeated several fields, especially urology [24]. The widespread adoption of robotics worldwide has led to an increasing body of evidence supporting its noninferiority to open surgery and its benefits for both surgeons and patients for selected intervention [25, 26].

Thus, the transplantation community has been hesitant to such change, and OKT still remains the gold standard approach at most center worldwide [21].

In recent years, several teams have developed and standardized the technique of RAKT, aiming to reduce the morbidity of kidney especially in the obese population [27]. Our results confirm that robotic approach is safe for living donor kidney transplantation in obese recipients. Indeed, there are consistent with published literature [26, 28–30].

The study by Campi [26], which assessed the non-inferiority of robot-assisted versus open kidney transplantation from deceased donors, reported comparable postoperative complication rates of 7% in both groups. After a median follow-up of 31 months, overall survival and dialysis-free survival were also similar between the two approaches.

Furthermore, a systematic review including 2136 patients [29] demonstrated postoperative complication rates of 26% in the open surgery group and 17.8% in the robot-assisted group. The incidence of delayed graft function was 4.9% versus 2.3% in the robotic group. Mid-term functional outcomes, patient survival, and graft survival were comparable between the two techniques.

In our study, the rate of postoperative complications was similar between the two groups. However, the rate of tardive reoperation was significantly higher in the open surgery group, particularly for eventration. This suggests that robot-assisted procedures may be associated with a lower risk of postoperative complications, which is particularly relevant in patients with obesity, who are known to be at higher risk.

However, the present study is not devoid of limitations. First, this is a retrospective non-randomized study with potential selection bias. Second, due to its single-institution nature, our results may not be generalizable to all clinical scenarios. Our study includes a small number of operating surgeons. The limited sample size (*n* = 86) represents an inherent limitation of the present study and may reduce its statistical power to identify differences in infrequent early postoperative complications, such as Clavien–Dindo grade ≥ 3 events.

This study adds evidence supporting the use of minimally invasive techniques in kidney transplantation as equivalent to traditional open approaches in terms of graft survival and patient survival, and potentially superior in terms of post-operative morbidity.

## Conclusions

This study confirms the safety of the robotic approach in kidney transplantation in the setting of obese recipients. The combination of reduced post-operative surgical reintervention after POD 90 and equivalent mid-term functional outcomes encourage the use of robotic-assisted approach in this specific population.

## Data Availability

No datasets were generated or analysed during the current study.

## Abbreviations

BMI: Body mass index
DGF: Delayed graft function
eGFR: Estimated glomerular filtration rate
KT: Kidney transplantation
LDN: Living-donor nephrectomy
LOH: Length of hospitalization
MRA: Multiple renal arteries
OKT: Open kidney transplantation
POD: Post-operative day
RAKT: Robotic-assisted kidney transplantation

## Footnotes

1 p<0.005
